# Supplementary material for: Characterization of Male Flower Induction by Silver Thiosulfate Foliar Spray in Female Cannabis at the Middle Reproductive Stage for Breeding
Source: Plants (Basel). 2024 Aug 30;13(17):2429. doi: 10.3390/plants13172429 (PMC11397453; doi:10.3390/plants13172429)
Supplement: Supplementary file 1 [file plants-13-02429-s001.zip › plants-3123925-supplementary.pptx]

## Slide 1
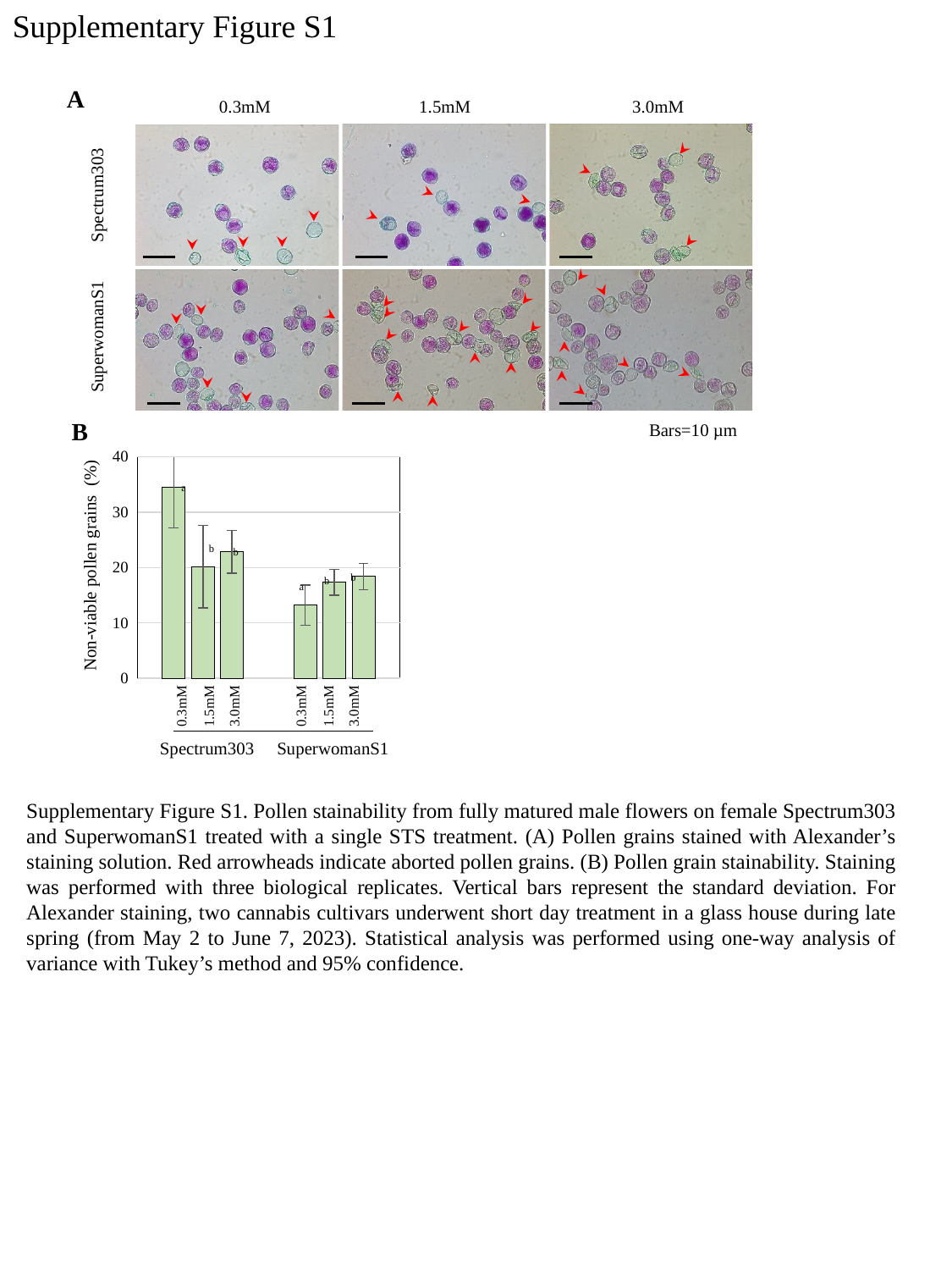

Supplementary Figure S1
A
0.3mM
1.5mM
3.0mM
Spectrum303
SuperwomanS1
B
Bars=10 µm
### Chart
| Category | 0.3mM | 1.5mM | 3mM |
|---|---|---|---|
| Spectrum303 | 34.535425992166886 | 20.148396993039057 | 22.83716182431642 |
| SuperwomanS1 | 13.214279943917198 | 17.33252846641734 | 18.371130841192993 |a
b
b
Non-viable pollen grains (%)
b
b
a
0.3mM
1.5mM
3.0mM
0.3mM
1.5mM
3.0mM
SuperwomanS1
Spectrum303
Supplementary Figure S1. Pollen stainability from fully matured male flowers on female Spectrum303 and SuperwomanS1 treated with a single STS treatment. (A) Pollen grains stained with Alexander’s staining solution. Red arrowheads indicate aborted pollen grains. (B) Pollen grain stainability. Staining was performed with three biological replicates. Vertical bars represent the standard deviation. For Alexander staining, two cannabis cultivars underwent short day treatment in a glass house during late spring (from May 2 to June 7, 2023). Statistical analysis was performed using one-way analysis of variance with Tukey’s method and 95% confidence.

## Slide 2
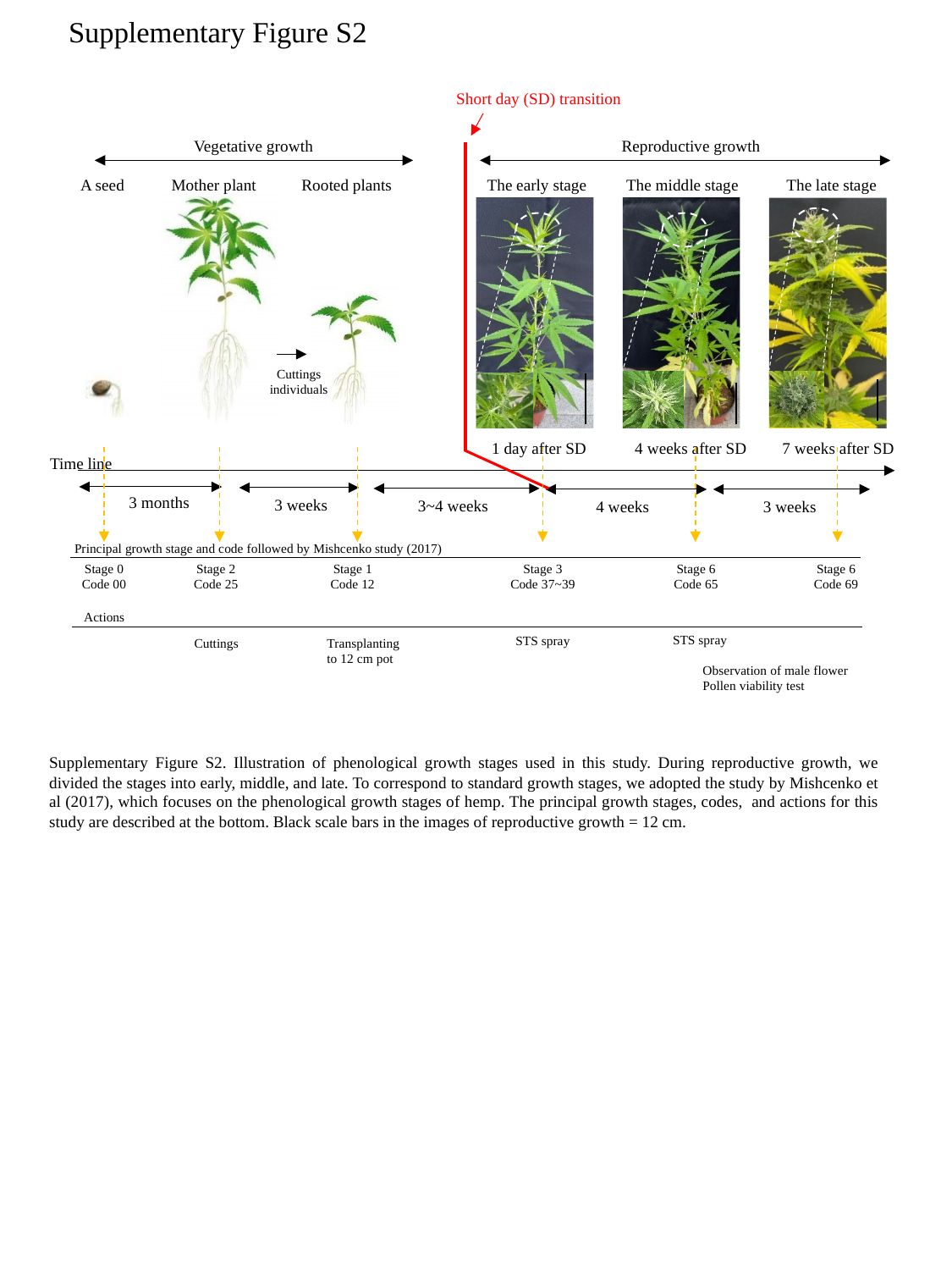

Supplementary Figure S2
Short day (SD) transition
Vegetative growth
Reproductive growth
A seed
Mother plant
Rooted plants
The early stage
The middle stage
The late stage
Cuttings
individuals
1 day after SD
4 weeks after SD
7 weeks after SD
Time line
3 months
3 weeks
3~4 weeks
4 weeks
3 weeks
Principal growth stage and code followed by Mishcenko study (2017)
Stage 0
Code 00
Stage 2
Code 25
Stage 1
Code 12
Stage 3
Code 37~39
Stage 6
Code 65
Stage 6
Code 69
Actions
STS spray
STS spray
Cuttings
Transplanting
to 12 cm pot
Observation of male flower
Pollen viability test
Supplementary Figure S2. Illustration of phenological growth stages used in this study. During reproductive growth, we divided the stages into early, middle, and late. To correspond to standard growth stages, we adopted the study by Mishcenko et al (2017), which focuses on the phenological growth stages of hemp. The principal growth stages, codes, and actions for this study are described at the bottom. Black scale bars in the images of reproductive growth = 12 cm.
